# Synthesis and characterization of single-crystalline zinc tin oxide nanowires

**DOI:** 10.1186/1556-276X-9-210

**Published:** 2014-05-05

**Authors:** Jen-Bin Shi, Po-Feng Wu, Hsien-Sheng Lin, Ya-Ting Lin, Hsuan-Wei Lee, Chia-Tze Kao, Wei-Hsiang Liao, San-Lin Young

**Affiliations:** 1 Department of Electronic Engineering, Feng Chia University, 100, Wen-Hwa Rd, Seatwen, Taichung 40724, Taiwan; 2 Ph.D. Program of Electrical and Communications Engineering, Feng Chia University, 100, Wen-Hwa Rd, Seatwen, Taichung 40724, Taiwan; 3 Department of Dentistry, College of Oral Medicine, Chung Shan Medical University, No. 110, Sec. 1, Jianguo N. Rd, Taichung 40201, Taiwan; 4 Dental Department, Chung Shan Medical University Hospital, No. 110, Sec. 1, Jianguo N. Rd, Taichung 40201, Taiwan; 5 Master Degree Program in Institute of Oral Sciences, College of Oral Medicine, Chung Shan Medical University, No. 110, Sec. 1, Jianguo N. Rd, Taichung 40201, Taiwan; 6 Department of Electronic Engineering, Hsiuping University of Science and Technology, Taichung 41280, Taiwan

**Keywords:** Zinc tin oxide, Nanowires, AAO membrane

## Abstract

Crystalline zinc tin oxide (ZTO; zinc oxide with heavy tin doping of 33 at.%) nanowires were first synthesized using the electrodeposition and heat treatment method based on an anodic aluminum oxide (AAO) membrane, which has an average diameter of about 60 nm. According to the field emission scanning electron microscopy (FE-SEM) results, the synthesized ZTO nanowires are highly ordered and have high wire packing densities. The length of ZTO nanowires is about 4 μm, and the aspect ratio is around 67. ZTO nanowires with a Zn/(Zn + Sn) atomic ratio of 0.67 (approximately 2/3) were observed from an energy dispersive spectrometer (EDS). X-ray diffraction (XRD) and corresponding selected area electron diffraction (SAED) patterns demonstrated that the ZTO nanowire is hexagonal single-crystalline. The study of ultraviolet/visible/near-infrared (UV/Vis/NIR) absorption showed that the ZTO nanowire is a wide-band semiconductor with a band gap energy of 3.7 eV.

## Background

In recent years, one-dimensional (1D) nanostructures, such as nanotubes, nanowires, nanorods, nanobelts, nanocables, and nanoribbons, have stimulated considerable interest for scientific research due to their physical and chemical properties. The zinc tin oxide (ZTO) nanostructures in particular show promising results in electronics, magnetics, optics, etc., and may have great potential for application in the next generation of nanodevices. Anodic aluminum oxide (AAO) membrane-based assembling has been widely applied in recent years to produce nanowires with extremely long length and a high aspect ratio and to provide a simple, rapid, and inexpensive way for fabricating nanowires as aligned arrays [1-3].

Zn-Sn-O (ZTO) is an interesting semiconducting material with a band gap energy (*E*_g_) of 3.6 eV [4,5]. It has demonstrated great potential for application in various areas, such as transparent conducting oxides used as photovoltaic devices, flat panel displays, solar cells, and gas sensors, due to its high electron mobility, high electrical conductivity, and low visible absorption [4-7].

Over the past decades, many research efforts have been made on the preparation of ZTO films. Recently, there have been very few references for our knowledge about ZTO. For ZTO nanowires, in a previous research, transparent semiconducting ternary oxide Zn_2_SnO_4_ nanowires were synthesized by the thermal evaporation method without any catalyst [8]. A mixture of SnO and ZnO powder was placed into a small ceramic boat, which was positioned at the center of a quartz tube. The temperature of the system was increased to 875°C and kept at this temperature for 30 min. Additionally, single-crystalline ZTO nanowires were prepared using a simple thermal evaporation method [9]. A mixture of Zn and Sn powders (10:3 weight ratio) was used as the source material, and the whole experiment was performed in a horizontal tube furnace. The temperature at the tube center increased at a constant rate of 25°C/min from room temperature to reaction temperature (approximately 800°C), where it was then maintained for 90 min. During that period, metal powders were heated, vaporized, transported along the Ar flow, and finally deposited on the substrates to form the ZTO nanowires through reaction. Moreover, mixed oxide ZnO-Zn_2_SnO_4_ (ZnO-ZTO) nanowires with different sizes were prepared in a horizontal tube furnace by a simple thermal evaporation method [10]. Zn and SnO mixed powders (2:1 in molar ratio) were positioned in a ceramic boat, which was loaded into the center of the tube. The furnace was heated at a rate of 80°C/min up to and maintained at 800°C, 900°C, and 1,150°C for 30 min each, respectively. However, there have been a few reports on ZTO nanowires that have been fabricated with AAO membrane-assisted synthesis using electrodeposition and heat treatment methods.

In this study, we report the synthesis and characterization of ZTO (ZnO with heavy Sn doping of 33 at.%) nanowires based on highly ordered AAO membrane by an electrodeposition method. The microstructure and optical properties of ZTO nanowires are then discussed.

## Methods

The fabrication process contains three steps: (1) electrochemical formation of an AAO membrane with highly ordered hexagonal arrays of nanochannels, (2) electrochemical deposition of Zn-Sn alloy into the AAO membrane, and (3) oxidation of the Zn-Sn alloy nanowires with the AAO membrane in the furnace.

### Preparation of AAO template

The AAO membrane used in our experiment was prepared by a two-step anodization process as described previously [1-3]. Finally, the diameter of each nanochannel was about 60 nm.

### Preparation of ZTO nanowires

Before electrodeposition, a layer of Pt was sputtered on one side of the AAO membrane as a conductive layer. Zn-Sn alloy nanowires were electrodeposited in the AAO membrane under alternating current (AC; 10 V) and direct current (DC; 4 V) voltages within the solution containing ZnSO_4_ · 7H_2_O, SnSO_4_, and distilled water. The starting solution of synthesis of Zn-Sn alloy nanowires was a mixture solution of ZnSO_4_ · 7H_2_O and SnSO_4_ with a 2:1 molar ratio. The samples of Zn-Sn alloy nanowires in an AAO membrane were subsequently placed in a furnace that was heated from room temperature (heating rate 5°C/min) to 700°C and maintained for 10 h. After the reaction was terminated, the furnace was naturally cooled down to room temperature, and ZTO nanowires were completely form-ordered after oxidation.

### Characterization of ZTO nanowire

The morphologies of the as-prepared AAO membrane and the ZTO nanowires were analyzed by field emission scanning electron microscopy/energy dispersive spectrometery (FE-SEM/EDS; Hitachi S-4800, Hitachi, Ltd., Tokyo, Japan). The crystal structure of the nanowires was examined by X-ray diffraction (XRD; Shimadzu XRD-6000, Shimadzu Corporation, Kyoto, Japan) utilizing Cu Kα radiation. More details about the microstructure of the ZTO nanowires were investigated by the high-resolution transmission electron microscopy/corresponding selected area electron diffraction (HR-TEM/SAED; JEOL JEM-2010, JEOL Ltd., Tokyo, Japan). After the ZTO nanowires were absolutely dispersed in distilled water using a supersonic disperser, the absorption spectra of the ZTO nanowires were measured on an ultraviolet/visible/near-infrared (UV/Vis/NIR) spectrophotometer (Hitachi U-3501).

## Results and discussion

For the AC process, the alternation of the electric field will remove the undesired deposition that is deposited on the surface of the AAO membrane. For the DC process, the direction of the electric field will result in a high density and high-quality deposition to form highly ordered Zn-Sn alloy nanowires (not shown). Therefore, we have selected appropriate AC (10 V) and DC (4 V) voltages to prepare high-quality nanowires.

### Morphology of AAO template and ZTO nanowires

**Figure 1:**
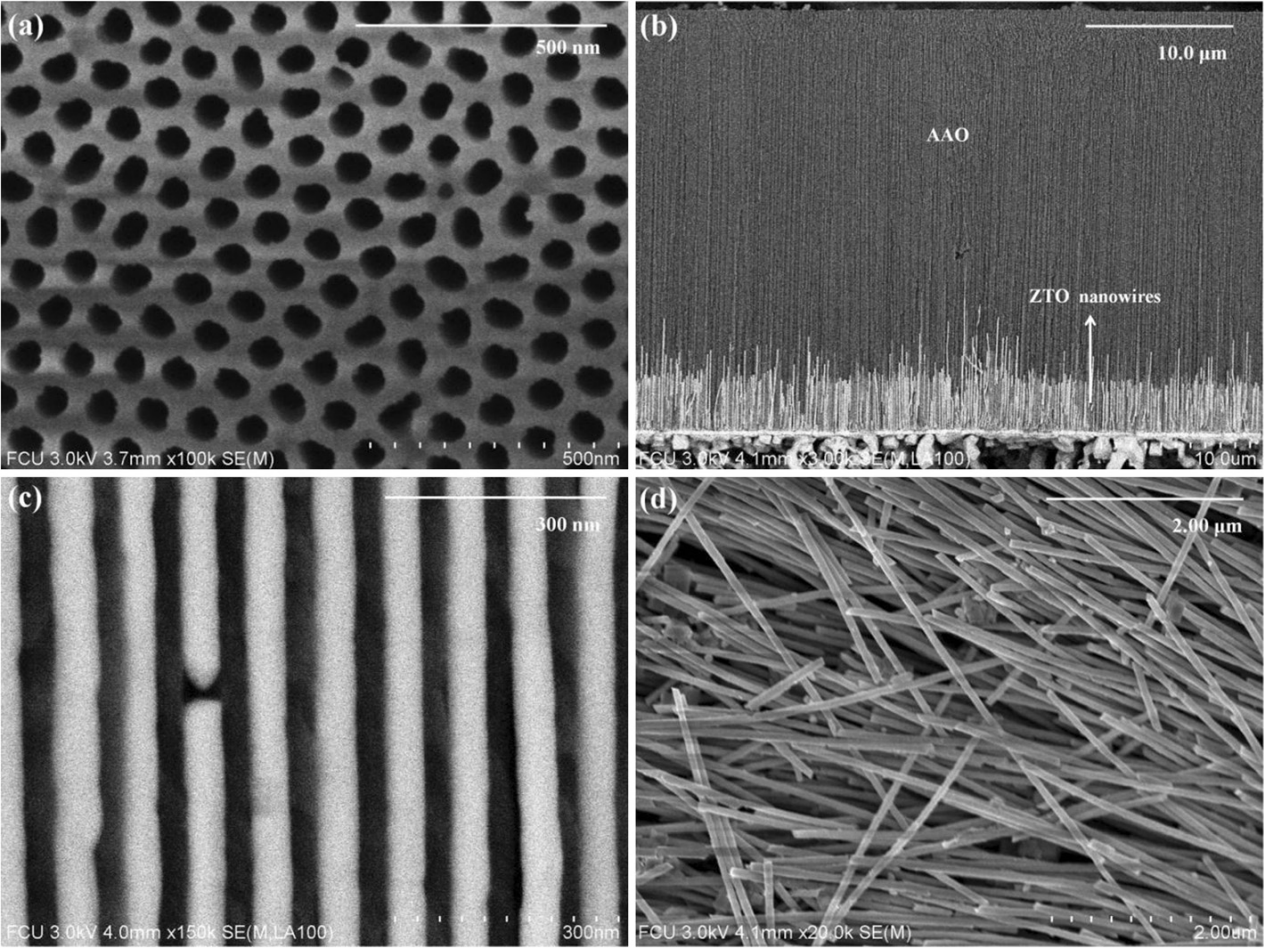
**FE-SEM images of the AAO template and ZTO nanowires. (a)** Top view. **(b)** Cross-sectional view of ZTO nanowires with a pore diameter of about 60 nm oxidized at 700 °C for 10 h in the AAO membrane. **(c)** Cross-sectional view at high magnification. **(d)** The AAO membrane was absolutely dissolved by NaOH solution.

The morphology of the as-synthesized product was examined by FE-SEM. Figure 1 shows a morphological image of the AAO template and ZTO nanowires.

The typical FE-SEM image (Figure 1a) shows that the surface of the AAO membrane was still kept clean and had no deposition after the ZTO nanowires were oxidized at 700 °C for 10 h The image also shows that the pores on the AAO membrane have a uniform size and are arranged in a hexagonal honeycomb structure. Figure 1b shows a cross-sectional FE-SEM image of the ZTO nanowires embedded in the porous AAO membrane. It is obvious that the ZTO nanowires in the AAO membrane are well aligned, and the length is about 4 μm. Figure 1c reveals a cross-sectional FE-SEM image of the ZTO nanowires at high magnification. It is clear that these nanowires are parallel to each other, and they have a very high aspect ratio. After thoroughly dissolving the AAO membrane by NaOH etching, followed by rinsing with distilled water, the ZTO nanowires are still on the substrate surface. Figure 1d shows the FE-SEM image of the as-prepared ZTO nanowires with a diameter of about 60 nm without the AAO membrane. As observed from this figure, large-scale ZTO nanowires were obtained.

However, the EDS spectrum of the ZTO nanowires is not shown. EDS quantitative analysis revealed that these nanowires are composed of zinc, tin, and oxygen, which is in effective conformity with the XRD results. In this study, the atomic ratio of the Zn/(Zn + Sn) composition is close to 0.67 of ZTO nanowires, indicating that the ZTO nanowires were well crystallized and in good conformity with the Zn/(Zn + Sn) molar ratio of a starting solution of 2:3. The co-electrodeposition technique (Zn and Sn) offers simple and flexible control of the ZTO nanowire composition. This method is excellent for good-quality ZTO nanowire synthesis. Most importantly, co-depositing the Zn and Sn alloy nanowires to create the ZTO nanowires on the AAO template has the advantage that the content of Zn/Sn is comparatively easy to control.

### Crystal structures of ZTO nanowires

**Figure 2:**
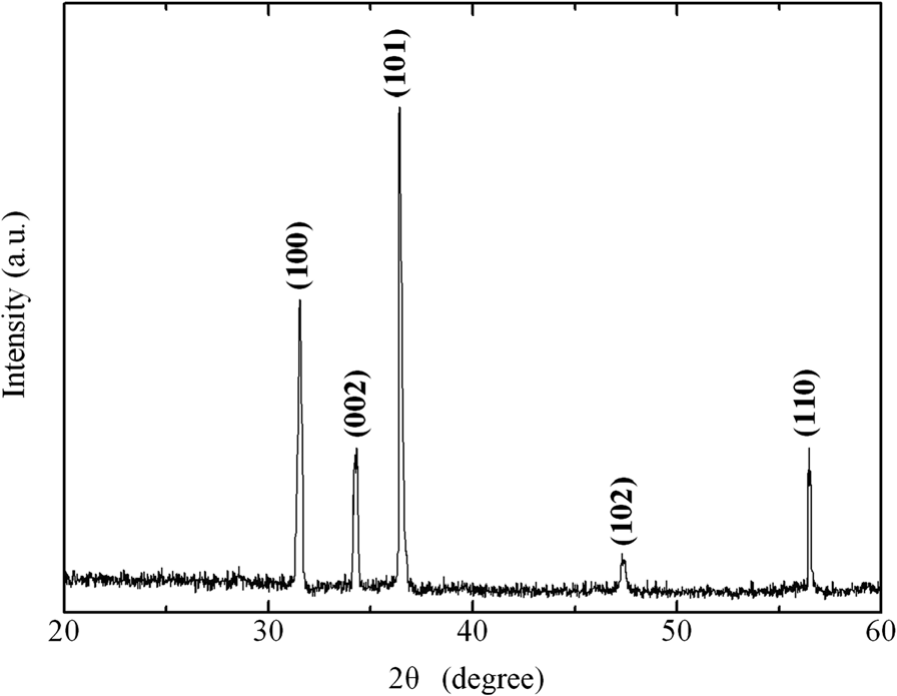
X-ray diffraction patterns as-prepared of ZTO nanowires without an AAO membrane.

The structure analysis of the as-synthesized product was carried out by XRD. Figure 2 shows the XRD patterns of ZTO nanowires with 60-nm diameter without an AAO membrane.

After heat treatment at 700°C for 10 h, all of the Zn and Sn peaks disappeared, indicating that the Zn and Sn deposited in the channels of AAO had been completely oxidized. In addition, the peak positions and their relative intensities are consistent with the existing literature data for pure ZnO (JCPDS card file, no. 80-0075). In our experiment, the heat treatment method was used to prepare the ZTO nanowires. In the process of heat treatment, well-crystallized ZTO nanowires with good uniformity can be synthesized at 700°C for 10 h. Detailed information on the microstructure of as-prepared ZTO nanowires was obtained by HR-TEM.

**Figure 3:**
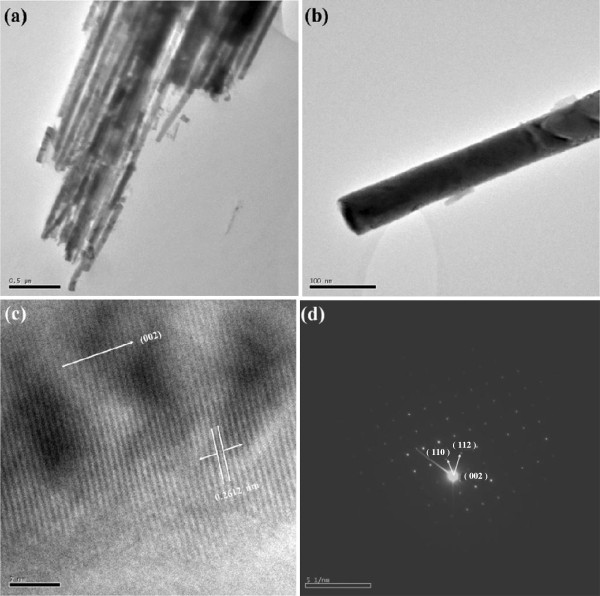
**HR-TEM images and SAED pattern of ZTO nanowires. (a)** The low-magnification HR-TEM image of ZTO nanowires. **(b)** The high-magnification HR-TEM image of an individual ZTO nanowire. **(c)** SAED pattern of an individual ZTO nanowire. **(d)** HR-TEM image of a single ZTO nanowire with lattice fringes.

A low-magnification HR-TEM image (Figure 3a) illustrates the numerous ZTO nanowires. Figure 3b reveals the HR-TEM image of an individual ZTO nanowire. The diameter of the nanowire is about 60 nm. The lattice spacing is approximately 0.2612 nm, corresponding to the (002) plane of ZTO (Figure 3c). Figure 3d is a typical SAED pattern taken from an individual nanowire. The SAED pattern reveals that the nanowire is a single-crystalline hexagonal structure growing along the *c*-axis, i.e., in the (002) direction.

### Optical properties of ZTO nanowires

**Figure 4:**
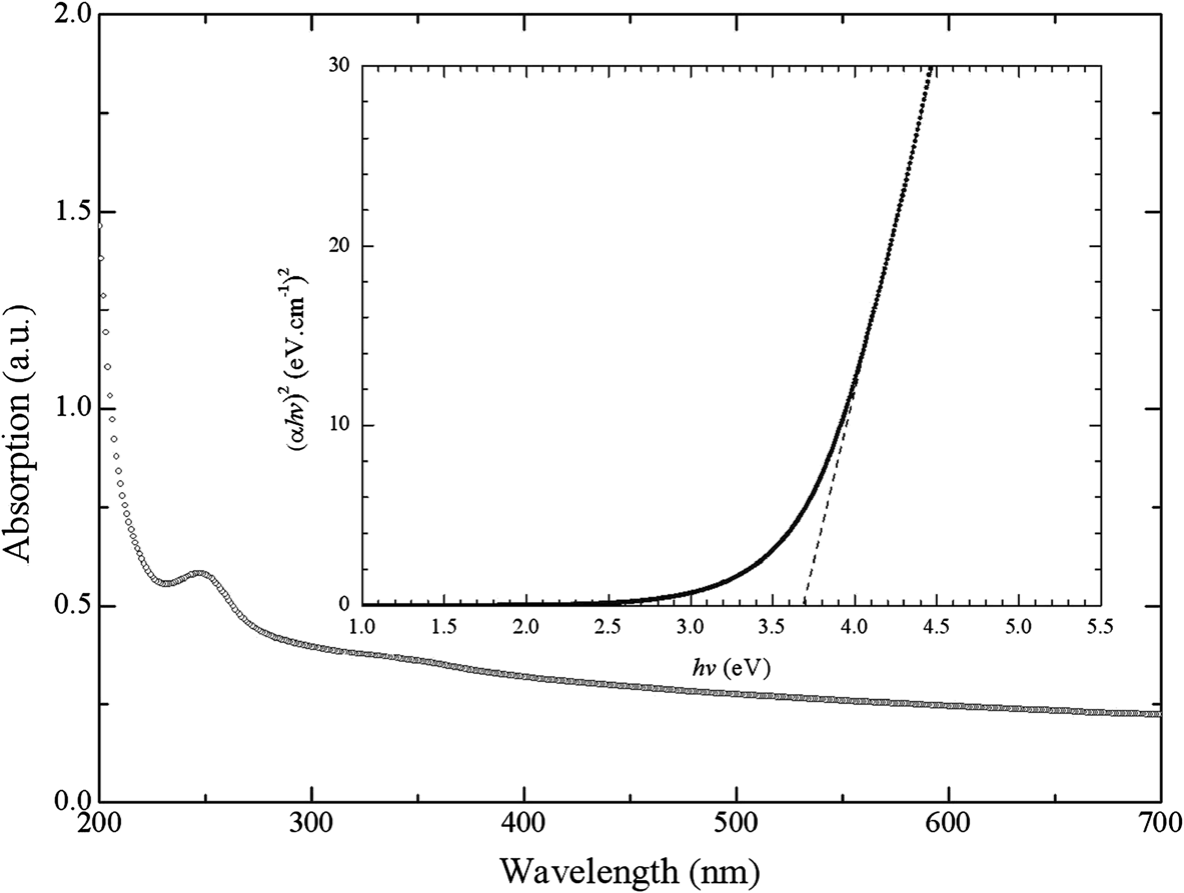
**UV/Vis/NIR absorption spectra with ZTO nanowires and (*αhν*)**^**2 **^**versus *hν *** **plot (inset).**

UV/Vis/NIR absorption spectra of samples were recorded in an airtight environment at room temperature with a wavelength range of 200 to 700 nm. Figure 4 shows the optical absorption spectra of the ZTO nanowires.

It can be observed that these nanowires have an absorption peak of around 250 nm. Young et al. showed that ZTO thin films were grown by RF magnetron sputtering onto glass substrates [11]. They observed a strong absorption of the ZTO film at 350 nm with a grain diameter of about 100 nm. Other research works have shown that nanosized ZTO particles are synthesized by a simple hydrothermal process in a water/ethylene glycol mixed solution using amines (ethylamine, *n*-butylamine, *n*-hexylamine, and *n*-octylamine) as a mineralizer [12]. The grain size of ZTO hexagonal particles varied in that experiment in the range of 40 to 70 nm and had an absorption peak of around 250 nm. However, our value of absorption peak is smaller than the value of films (350 nm) and is consistent with the value of nanoparticles (250 nm). Our value of absorption peak is reasonable and is consistent with the results found in other research works [11,12].

In order to determine the nature of the band gap of the nanostructured material, either indirect or direct, the spectral behavior near the fundamental absorption edge can be calculated by considering the following expression of the absorption coefficient (*α*) versus photon energy (*hν*) [13]:

(1) $$\mathrm{\alpha h\nu}=A{\left(\mathrm{h\nu}-E_{g}\right)}^{n}$$

where *hν* is the photon energy and *E*_*g*_ is the optical band gap corresponding to transitions indicated by the value of *n*. In particular, *n* is 1/2, 3/2, 2, and 3 for direct allowed and forbidden transitions, and indirect allowed and forbidden transitions, respectively. Factor *A* is the constant having separate values for different transitions. When the linear portion of the graph is extrapolated to zero, the intercept of the *hν* axis gives the optical band gap (*E*_*g*_). The (*αhν*)^2^ versus *hν* plot is shown in the inset in Figure 4. This plot is known as a Tauc plot. The analysis of the absorption spectrum obtained for our samples shows that the spectral variation of the absorption coefficient that is within the fundamental absorption region can be fitted by Equation 1. However, when *n* = 3/2, 2, and 3, the band gap energies were found to be a negative number, which is not physically reasonable.

The inset in Figure 4 shows the (*αhν*)^2^ against *hν* plot. The absorption spectra of ZTO nanowires as *n* = 1/2, which is the allowed direct transition for these nanowires, fit the relationship of (*αhν*)^2^. In this inset figure, we observed that the curve has an obvious straight line fit from 4.0 to 4.5 eV. This result indicates that the optical energy gap is a direct transition. The band gap energy (*E*_*g*_) of ZTO nanowires with a diameter of about 60 nm is estimated to be 3.7 eV as *n* = 1/2 for extrapolation. Nanocrystals of ZTO were synthesized by the hydrothermal method [14]. A mixture of ZnSO_4_ · 6H_2_O and SnCl_4_ · 5H_2_O was used as the starting material that was then dissolved into distilled water. The NaOH solution was then dropped into the above solution under magnetic stirring for 15 min. The resulting precipitates were collected by centrifugation at 3,000 rpm, thoroughly rinsed with distilled water and ethanol, and dried at 80°C in an oven for 5 h. The particle sizes of ZTO nanocrystals were calculated to be about 100 to 150 nm. The optical band gaps of various ZTO nanocrystals were between 3.69 and 3.73 eV. In addition, ZTO nanoparticles were synthesized by the hydrothermal process [12].

In a previous study, ZnCl_2_ and SnCl_4_ · 5H_2_O were added to a water/ethylene glycol solvent under magnetic stirring. Then, an *n*-butylamine aqueous solution was then dropped into the solution and stirred for 0.5 h. Finally, the product was dried in air at 60°C for 10 h. The as-prepared ZTO nanoparticles had a band gap of 3.7 eV. Moreover, single-crystalline ZTO nanorods were prepared by the hydrothermal process with the use of hydrazine hydrate as an alkaline mineralizer instead of NaOH or NH_3_ · H_2_O [15]. Previous studies created a product consisting of rod-like nanostructures of 2 to 4 nm in diameter, called 5-nm ZTO nanorods. The optical band gap of the nanorods was found to be 3.87 eV. Consequently, the band gap energy of ZTO nanowires in this study is between the smallest band gap energy (3.69 eV) and the largest band gap energy (3.87 eV). This band gap energy of ZTO nanowires is reasonable with references [12-15]. ZTO thin films have been widely used in fabricating semiconductor gas sensors [16,17]. Yet, gas sensors prepared from 1D nanostructure ZTO have rarely been reported. To our knowledge, because of its high surface-to-volume ratio, the 1D nanostructure is more sensitive than the thin film material. Through electrochemical deposition and oxidation methods, we will develop ZTO nanowires that can be applied in a gas sensor.

## Conclusion

Highly ordered ZTO nanowires with heavy tin doping (approximately 1/3) embedded in the AAO membrane have been successfully fabricated by an electrodeposition and heat treatment method. The pure metal Zn and Sn were electrodeposited into the AAO membrane, which is measured to be 60 nm. ZTO nanowires can be synthesized by oxidizing the Zn-Sn alloy nanowires in the furnace at 700°C for 10 h. FE-SEM micrographs show that ZTO nanowires are dense, have uniform diameter, and are arranged parallel to each other. XRD analysis indicates that the ZTO nanowires have a hexagonal structure. The obtained ZTO nanowires with a Zn/(Zn + Sn) atomic ratio of 0.67 (approximately 2/3) were nearly the same as the Zn/(Zn + Sn) molar ratio of the starting solution (2:3). It can be said that the composition of ZTO nanowires can be strongly controlled by adjusting the Zn/Sn molar ratio in the starting solution through co-electrodeposition. The analysis of the HR-TEM/SAED results reveals the that ZTO nanowire is single-crystalline. The band gap of ZTO nanowires (3.7 eV) shows a direct transition and exhibits a linear relationship at 4.0 to 4.5 eV.

## Competing interests

The authors declare that they have no conflict of interest.

## Authors' contributions

J-BS conceived and designed the experiments and took part in the discussions and interpretation of the results; he also supervised the research performed by students. P-FW carried out the experiments, performed data analysis, and participated in the discussions. H-SL participated in the discussions and interpretation of the results. Y-TL carried out the experiments, performed data analysis, and took part in the discussions and interpretation of the results. H-WL, C-TK, W-HL, and S-LY participated in the discussions. All authors read and approved the final manuscript.

## Authors' information

J-BS is a professor in the Department of Electronic Engineering at Feng Chia University. P-FW, H-SL, Y-TL, and H-WL are PhD students of the Department of Electrical and Communications Engineering at Feng Chia University. C-TK is a professor in the Department of Dentistry at Chung Shan Medical University. W-HL is a master student in Institute of Oral Sciences at Chung Shan Medical University. S-LY is a professor in the Department of Electronic Engineering at Hsiuping University of Science and Technology.
